# Scleritis following intravitreal brolucizumab injection: a case series

**DOI:** 10.1186/s13256-024-04402-9

**Published:** 2024-02-29

**Authors:** Takuya Takayama, Satoru Inoda, Hidenori Takahashi, Rika Tsukii, Hana Yoshida, Yuka Kasuya, Kosuke Nagaoka, Ryota Takahashi, Yusuke Arai, Hidetoshi Kawashima

**Affiliations:** Department of Ophthalmology, Jichi Medical University, 3311-1 Yakushiji, Shimotsuke-Shi, Tochigi, 329-0431 Japan

**Keywords:** Brolucizumab, Scleritis, Intraocular inflammation, Case report

## Abstract

**Background:**

This study reports the first cases of scleritis following intravitreal brolucizumab (IVBr) injection for nAMD, emphasizing the need to be aware of the possibility of scleritis following IVBr injections.

**Case presentation:**

Case 1. A 74-year-old Japanese man with nAMD complained of conjunctivitis and decreased vision in the right eye 8 days after his eighth IVBr injection. Examination revealed scleritis without anterior inflammation. Topical 0.1% betamethasone and 0.3% gatifloxacin eye drops were started. The scleritis worsened in the following 2 weeks and became painful. He underwent sub-Tenon’s capsule triamcinolone acetonide (STTA) injection. Two days later, he returned with a complaint of severe vision loss. Fundus examination revealed retinal artery occlusion, vasculitis, and vitreous opacity in the right eye. Vitreous surgery was performed. Case 2. An 85-year-old Japanese woman with nAMD in the right eye complained of reddening of the eye 27 days after her fifth IVBr injection. Examination showed conjunctivitis and scleritis without anterior inflammation in the right eye. She was started on 0.1% fluorometholone and 0.5% levofloxacin hydrate eye drops. The scleritis worsened in the following 3 weeks. Her treatment was switched to 0.1% betamethasone eye drops. One month later, the scleritis had improved and a sixth IVBr injection was administered. There was no worsening of the scleritis at that time. However, 1 month after a seventh IVBr injection, she complained of severe hyperemia and decreased vision. Fundus examination revealed vitreous opacification. She underwent STTA, and the vitreous opacity improved in 24 days. Case 3. A 57-year-old Japanese man with nAMD complained of pain and decreased vision in the right eye 21 days after a fourth IVBr injection. Examination revealed scleritis with high intraocular pressure but no anterior chamber or fundus inflammation. STTA and topical eye drops were performed. One month later, scleritis improved but visual acuity didn’t due to progression of nAMD.

**Conclusions:**

Intraocular inflammation following IVBr injection may progress to the posterior segment. Scleritis can occur after IVBr injection, and topical eye drops alone may not be sufficient for initial treatment. Clinicians should consider the possibility of scleritis in patients with worsening inflammation after IVBr injection.

## Background

Age-related macular degeneration is a progressive disease and one of the main causes of blindness and decreased vision in the elderly [1]. Brolucizumab is an anti-vascular endothelial growth factor agent that was recently approved as a treatment for neovascular age-related macular degeneration (nAMD). Brolucizumab is expected to have a more prolonged effect than previous treatments and to reduce the treatment burden in patients with nAMD [2]. However, in the Phase III HAWK and HARRIER trials, intraocular inflammation (IOI) was reported to be more common in patients with nAMD who received brolucizumab than in those who received aflibercept [3, 4]. Reports of the incidence of IOI following IVBr injection that requires prompt discontinuation and anti-inflammatory therapy range from 2.4 to 19% [4–7]. IOI following IVBr injection includes uveitis, iritis, vitreous opacity, retinal vasculitis, and retinal artery occlusion [8–10]. However, to our knowledge, there have been no reports of scleritis following IVBr injection. We have treated three Japanese patients who developed scleritis following IVBr injection for nAMD.

## Case presentation

### Case 1

**Fig. 1:**
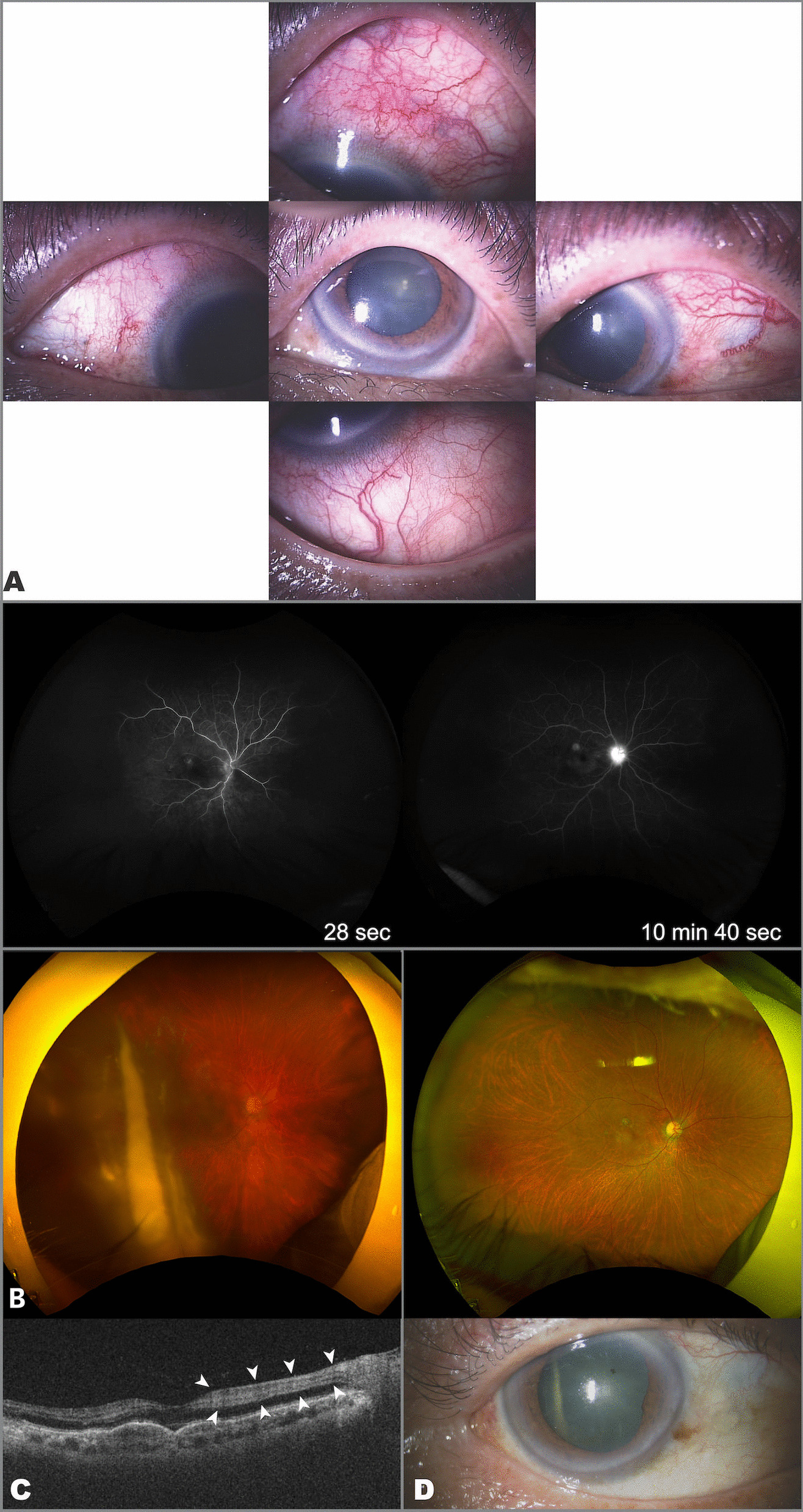
Clinical findings in Case 1. **A** Significant upper scleritis was observed in the right eye after the eighth intravitreal brolucizumab injection. **B** Fundus angiography shows delayed inflow and peripheral arterial occlusion. Fundus photograph of the right eye shows vitreous opacity and retinal whitening in the upper and temporal macular. **C** Retinal artery occlusion has occurred, and slight hyperreflectivity of the inner nuclear layer is seen in the macula (white arrow). **D** Scleritis and vitreous opacity improved

The patient was a 74-year-old Japanese man who had received 11 intravitreal aflibercept injections for pachychoroid polypoidal choroidal vasculopathy in the right eye over a period of 3 years. His medical history included laparoscopic subtotal esophagectomy for esophageal cancer. There was no history of uveitis or retinal vascular occlusion. Eight days after his eighth IVBr injection, he complained of redness and decreased vision in the right eye (Fig. 1A). Examination revealed scleritis without anterior inflammation in the right eye with a slight increase in intraocular pressure to 24 mmHg but no decrease in visual acuity of 20/25. We diagnosed scleritis, for which he was prescribed 0.1% betamethasone and 0.3% gatifloxacin eye drops four times daily. Two weeks later (26 days after the last IVBr injection), the inflammation had worsened and become painful and intraocular pressure had increased to 27 mmHg. Examination revealed keratic precipitates and fibrin in the vitreous without vasculitis, we diagnosed IOI following IVBr injection. In view of the worsening IOI, he underwent sub-Tenon’s capsule triamcinolone acetonide (STTA) injection. Two days later (28 days after his most recent IVBr injection), he complained of severe vision loss. Fundus angiography shows delayed inflow and peripheral arterial occlusion. Fundus examination revealed retinal artery occlusion with vasculitis and vitreous opacity in the right eye (Fig. 1B). Optical coherence tomography of the right eye shows slight hyperreflectivity of the inner nuclear layer (Fig. 1C), and a decrease in visual acuity to 20/500. Vitreous surgery was performed. On examination 39 days later, the patient’s scleritis and vitreous opacity had resolved completely and his visual acuity had improved to visual acuity of 20/125 (Fig. 1D).

### Case 2

**Fig. 2:**
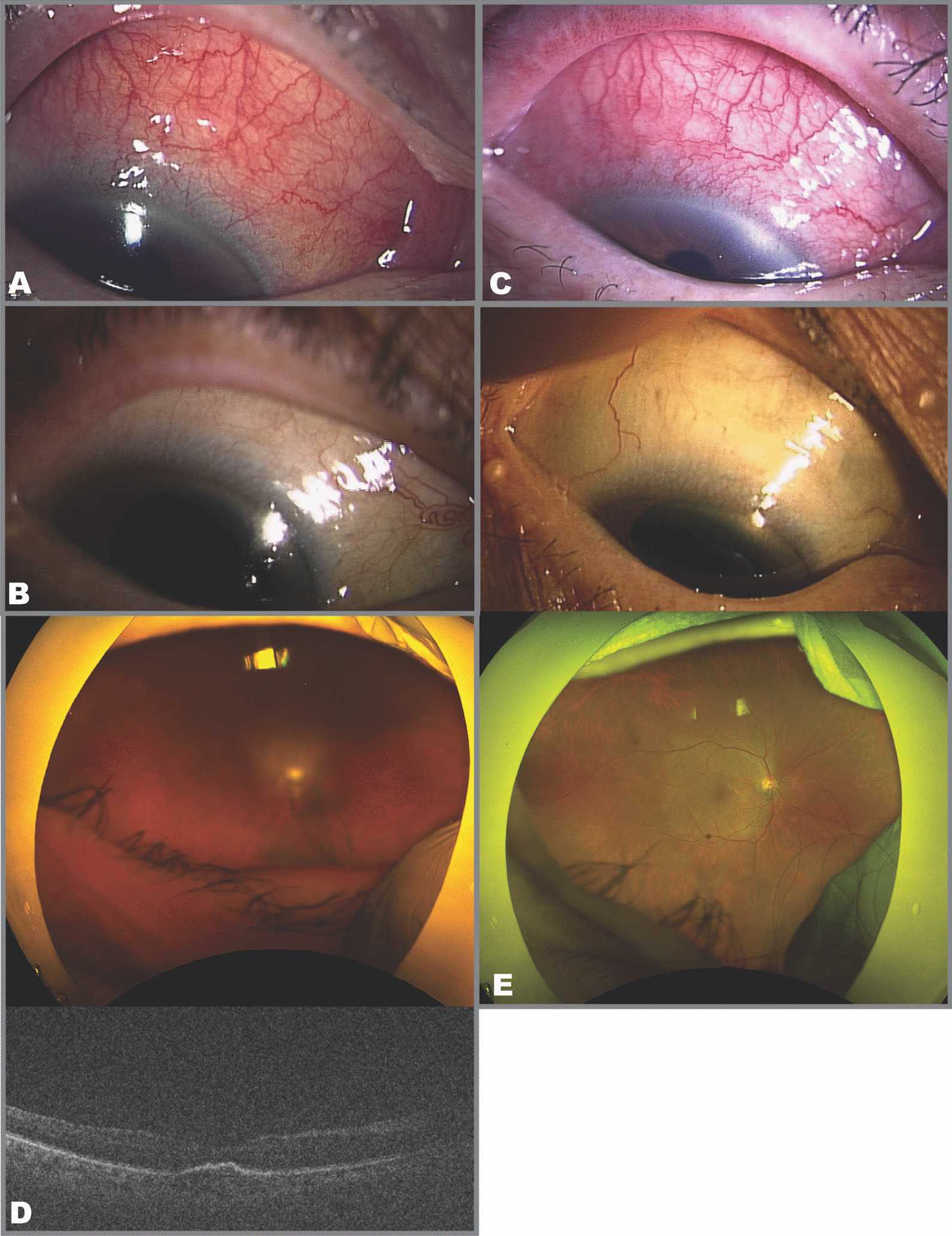
Clinical findings in Case 2. **A** Significant upper scleritis was observed in the right eye after the fifth intravitreal brolucizumab injection. **B** Scleritis improved after treatment with topical eye drops. **C** Upper scleritis worsened after the seventh intravitreal brolucizumab injection. **D** There was vitreous opacification but no retinal vasculitis or retinal vascular occlusion. The optical coherence tomography image was unclear because of vitreous opacification. **E** Scleritis and vitreous opacity improved

An 85-year-old Japanese woman with type 1 macular neovascularization in the right eye complained of redness in the affected eye 27 days after her fifth IVBr injection (Fig. 2A). She had hypertension. There was no history of any inflammatory or vascular disease of eye. Visual acuity decreased from 20/20 to 20/25, intraocular pressure was 21 mmHg. The eye showed scleritis without any sign of anterior chamber involvement. She was diagnosed, which was treated with 0.5% levofloxacin hydrate eye drops and 0.1% fluorometholone eye drops three times daily. After 16 days, she complained of hyperemia and heaviness in the eye. Visual acuity was 20/20 and intraocular pressure had increased further to 29 mmHg. Examination revealed slight anterior vitreous cells with scleritis and no anterior chamber inflammation. B-mode ultrasonography did not reveal any signs of posterior scleral thickening. She was started on topical tafluprost combined with timolol maleate eye drops as additional treatment. After 4 days (47 days after her most recent IVBr injection), scleritis did not show any inprovement, and the 0.1% fluorometholone eye drops were switched to 0.1% betamethasone eye drops. Seventy days after the most recent IVBr injection, the scleritis improved on the topical therapy (Fig. 2B). Subretinal fluid was seen in the macula at this time. She went on to receive a sixth IVBr injection, after which there was no worsening of her scleritis. Therefore, we did not think that her scleritis was IOI following IVBr at that time. However, a month after a seventh IVBr injection, she complained of worsening scleritis and loss of visual acuity (Fig. 2C). Ophthalmic examination revealed that her visual acuity had decreased to visual acuity of 20/1000 and the intraocular pressure had increased to 24 mmHg. Fundus examination revealed vitreous opacification without hemorrhage but no retinal vasculitis or retinal vascular occlusion (Fig. 2D). At this point, we diagnosed scleritis as IOI following IVBr and performed STTA injection. The vitreous opacity took 24 days to improve (Fig. 2E). By 87 days after STTA, her visual acuity had improved to 20/25 and intraocular pressure was 18 mmHg while on no topical therapy.

### Case 3

**Fig. 3:**
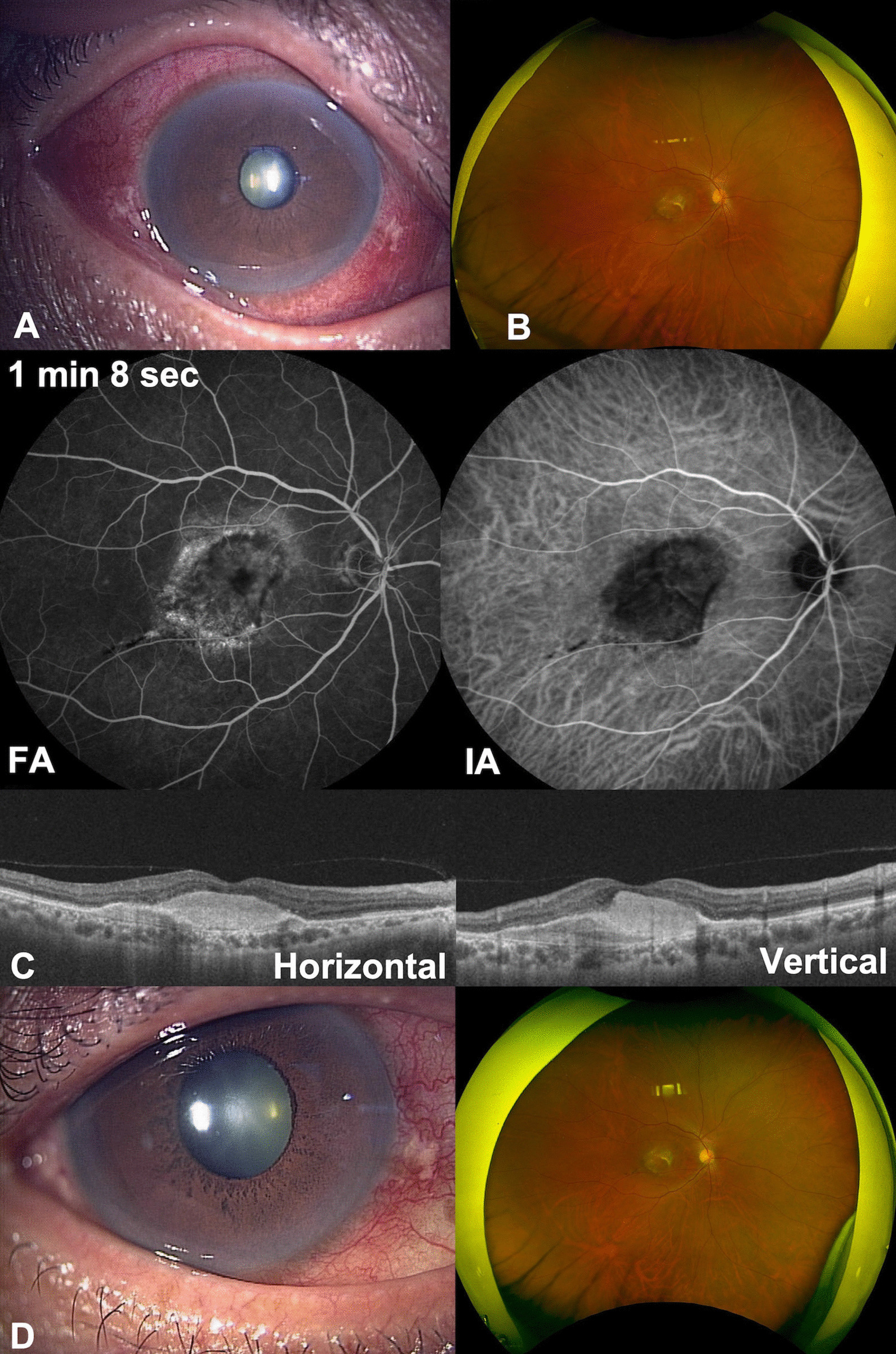
Clinical findings in Case 3. **A** Scleritis was observed after a fourth intravitreal brolucizumab injection. **B** Fundus images shows no vitreous opacity. **C** Fluorescein angiography and indocyanine green angiography did not show retinal vasculitis or retinal vascular occlusion. Optical coherence tomography showed fibrosis at the fovea centralis. **D** Scleritis gradually improved

A 57-year-old Japanese man with type 1 macular neovascularization in the right eye complained of pain and decreased vision in the affected eye 21 days after his fourth IVBr injection (Fig. 3A). His medical history included pancreatitis. His visual acuity decreased from 20/100 to 20/250 and his intraocular pressure was high at 49 mmHg. Examination revealed scleritis but no inflammation in the anterior chamber or fundus (Fig. 3B). Gonioscopy showed no abnormalities like angle nodules, peripheral anterior synechia, or angle neovascularization. He was diagnosed to have IOI following brolucizumab injection. He underwent STTA injection and received topical dorzolamide hydrochloride combined with timolol maleate and brimonidine tartrate eye drops. One week later, the scleritis had not resolved and intraocular pressure was still high at 38 mmHg; there was no inflammation or retinal arterial occlusion on fundoscopy (Fig. 3C). The patient was started on 0.1% betamethasone eye drops four times daily, with bimatoprost and ripasudil hydrochloride hydrate. One month later, the scleritis had improved and the intraocular pressure was normal to 14 mmHg, although the visual acuity remained at 20/250 because of progression of nAMD (Fig. 3D).

## Discussion and conclusions

We have reported three Japanese patients who developed scleritis after IVBr injections for nAMD. In all cases, the affected eyes initially presented without anterior inflammation nor posterior involvement. In the first two cases, we used only topical eye drops as initial treatment. However, after realizing that scleritis can occur following IVBr injection and that inflammation could progress to involve the posterior chamber, we performed STTA and intravitreal triamcinolone acetonide injections, and prescribed oral steroids with topical eye drops as in Case 3.

Although, on all three cases, blood tests and chest radiograph were performed as part of the scleritis investigation, causes like autoimmune disease such as rheumatoid arthritis, anti-neutrophil cytoplasmic antibody associated vasculitis, and systemic lupus erythematosus, infection such as tuberculosis, herpes simplex, varicella zoster virus, cytomegalovirus, human T-cell leukemia virus type 1, toxoplasma, and syphilis [11, 12], no clear cause could be identified. A possible contributing factor to brolucizumab-induced scleritis is the regurgitation of brolucizumab after vitreous injection [13]. One reason to support that the regurgitation is the cause is elevated intraocular pressure. In the extensive Intelligent Research In Sight registry cohort, elevated intraocular pressure was relatively rare, with scleritis being observed in approximately 5% of eyes. Conversely, anterior uveitis was more prevalent, presenting in about 14% of eyes [14]. A prior study identified potential risk factors, such as anterior chamber cells, for ocular hypertension in scleritis patients [15]. In this current series of cases, all the present cases had elevated intraocular pressure and redness with no anterior chamber cells, hypopyon, or fundus inflammation. Thus, the congestion of the precanalicular outflow pathway due to debris and cells may not be the primary factor. While the precise etiology of the pressure augmentation remains elusive in these cases, an increased scleral or episcleral vein pressure stemming from an immunological response to brolucizumab might be implicated.

This study has several limitations. In this research, B-mode ultrasonography was performed only for Case 2, and no clear signs of posterior scleral thickening were observed. For Cases 1 and 3, the presence or absence of posterior scleral thickening were unknown. In these three cases, the rise in intraocular pressure occurs without the concurrent presence of anterior chamber inflammation, which distinguishes it from the typical scleritis, which might represent the characteristics of scleritis following IVBr. When IOI occurs following IVBr injection, especially scleritis, eye drops alone might not be adequate initial treatment.

We have reported three cases of scleritis following IVBr injections. These cases suggest that ophthalmologists should be aware of the possibility of scleritis following IVBr injections. IOI following IVBr injections would get worsen even if the inflammation is only slight or within the anterior chamber. Topical treatment alone might not be adequate initial treatment for IOI following IVBr injections.

## Data Availability

Not applicable.

## Abbreviations

nAMD: Neovascular age-related macular degeneration
IOI: Intraocular inflammation
STTA: Sub-Tenon’s capsule triamcinolone acetonide
IVBr: Intravitreal brolucizumab injection
